# Anthropogenic modification shifts pollinator phenology in the eastern USA

**DOI:** 10.1098/rsos.260267

**Published:** 2026-09-09

**Authors:** Marina K. Marquis, Brittany M Mason, Bette Loiselle, Jacob S. Francis, Thomas Lilkendey, Janina Mulling, Corey T. Callaghan

**Affiliations:** ^1^ Department of Wildlife Ecology and Conservation, Fort Lauderdale Research and Education Center, Institute of Food and Agricultural Sciences, University of Florida, Davie, FL, USA; ^2^ Department of Biological Sciences, University of Florida, Gainesville, FL, USA; ^3^ School of the Environment, Coastal and Ocean Sustainability, Florida Atlantic University, Davie, Florida, USA

**Keywords:** anthropogenic modification, citizen science, insect pollinators, pollinator phenology

## Abstract

Understanding the effects of increasing anthropogenic changes on ecological communities is integral to predicting and mitigating such changes in the future. Insect pollinators are vital to ecosystem health. Quantifying pollinator phenology (i.e. flight period) offers insight into human impacts on ecosystem function, yet research is often constrained to narrow geographic and taxonomic scales. To address these constraints, we conducted a large-scale assessment of pollinator phenology across an anthropogenic modification gradient in the eastern USA using citizen science data from iNaturalist. We integrated 83 012 pollinator observations of 52 species with a remotely sensed layer of human modification to quantify species-specific differences in phenological change. We tested whether anthropogenic modification was a predictor of phenology. Our results reveal both taxonomic and spatial variation across a gradient of anthropogenic modification, with 19 species from three orders experiencing phenological shifts. We found evidence that pollinators in more anthropogenically modified areas of the eastern USA have seasons that start and end later in the year. By leveraging citizen science data, this study expands phenological research and provides a broader assessment across taxonomic and spatial scales.

## 1. Introduction

From 1993 to 2009, the human footprint increased by 9%, with 75% of the Earth's land surface experiencing anthropogenic modification [1]. Anthropogenic modification is broadly defined as any human-driven change to natural systems [1]. These human-driven changes cause habitat fragmentation, warmer temperatures and shifted resource availability, among other important changes that influence the structure and function of ecological communities [2]. For example, communities of reptiles [3], birds [4] and mammals [5] all shift because of anthropogenic modification. Anthropogenic modification also facilitates the establishment of non-native species, often resulting in ecological communities that are dominated by non-natives [6]. Although such changes typically decrease biodiversity [7–9], some species adapt to such changes and thrive in medium levels of anthropogenic habitat change (e.g. [10]). So, quantifying species-specific responses to anthropogenic change is important to understand how biodiversity patterns will shift in increasingly modified environments.

One such species-specific response to anthropogenic change is phenology—the timing of cyclical life-history events in an organism's life cycle (e.g. breeding, flowering and emergence). Phenology responds to climate change [11,12] across many diverse taxa, including birds [13], plants [14] and frogs [15]. Some flowering plants, for example, respond to anthropogenic variables, such as land use change, urbanization and global and urban warming, by shifting the time of first flowering and duration of flowering [16–20]. This shift in plant phenology and flowering periods may have cascading effects on insect pollinators, which may shift their phenology accordingly [21,22].

Insect pollinators exert a selective influence on angiosperm evolution, pollinate a significant proportion of angiosperms globally and are an abundant food source for higher trophic levels [23–26]. Therefore, insect pollinators are critical components of the environments they inhabit, and a useful lens through which to examine how these environments respond to anthropogenic change. With approximately 20-40% decline in insect pollinator populations [24], understanding insect responses to change is more important now than ever. For example, examining shifts in plant and pollinator phenology is key to revealing potential mismatches in plant-pollinator interactions, preserving ecosystem functions and informing conservation strategies in the face of increasingly anthropogenically modified environments. Although phenological shifts in the urban environment have been documented in insect pollinators [27–29], how their phenology shifts across taxonomic groups remains uncertain.

Owing to the logistical constraints of collecting insect data on a large scale, most insect phenology studies focus on only a few groups of pollinators. One of the most taxonomically expansive studies of urban insect pollinator phenology was conducted by [29]. They found that only three groups of pollinators—bumblebees, honeybees and small wild bees—experienced shifts in peak pollination times in urban areas of the Île-de-France region of France. Other pollinator groups (e.g. butterflies, large solitary bees, beetles, etc.) did not experience such shifts. However, this study, like most urban pollinator phenology studies, was conducted in a relatively small geographic area. In addition, comparison of these findings with results from other studies is difficult, as several similar studies focus solely on the phenology of bees and, therefore, cannot identify higher levels of taxonomic variation [27,28]. This study and studies like it also focus on urbanization rather than the broader effects of anthropogenic modification and are therefore limited in scope to urban centres. While these studies are invaluable, they miss out on large-scale phenology patterns, such as variation in the impact of anthropogenic change across spatial and taxonomic gradients. Such broad-scale patterns help shed light on where we might expect pollinators to have altered phenology and which pollinator groups are most likely to be affected, broadening the scope of our knowledge.

We aimed to expand upon the taxonomic and spatial limitations of previous work by using citizen science data. Citizen science, or community science, incorporates public participation in data collection, often expanding the potential geographic and taxonomic scale. Some studies use citizen science to overcome the logistical constraints of plant-pollinator research, with a relatively high accuracy of taxonomic identification of insects [21,30–32]. Our goal was to understand how pollinator phenology is associated with anthropogenic habitat modification across a diverse taxonomic group. Specifically, we aimed to (a) determine whether anthropogenic change is a significant predictor of insect pollinator phenology and (b) test whether pollinator phenological responses to anthropogenic change vary by taxonomic group. We predicted that the level of anthropogenic change would be a significant predictor of pollinator phenology and would vary across taxonomic groups.

## 2. Methods

### 2.1. Study region

**Figure 1.**
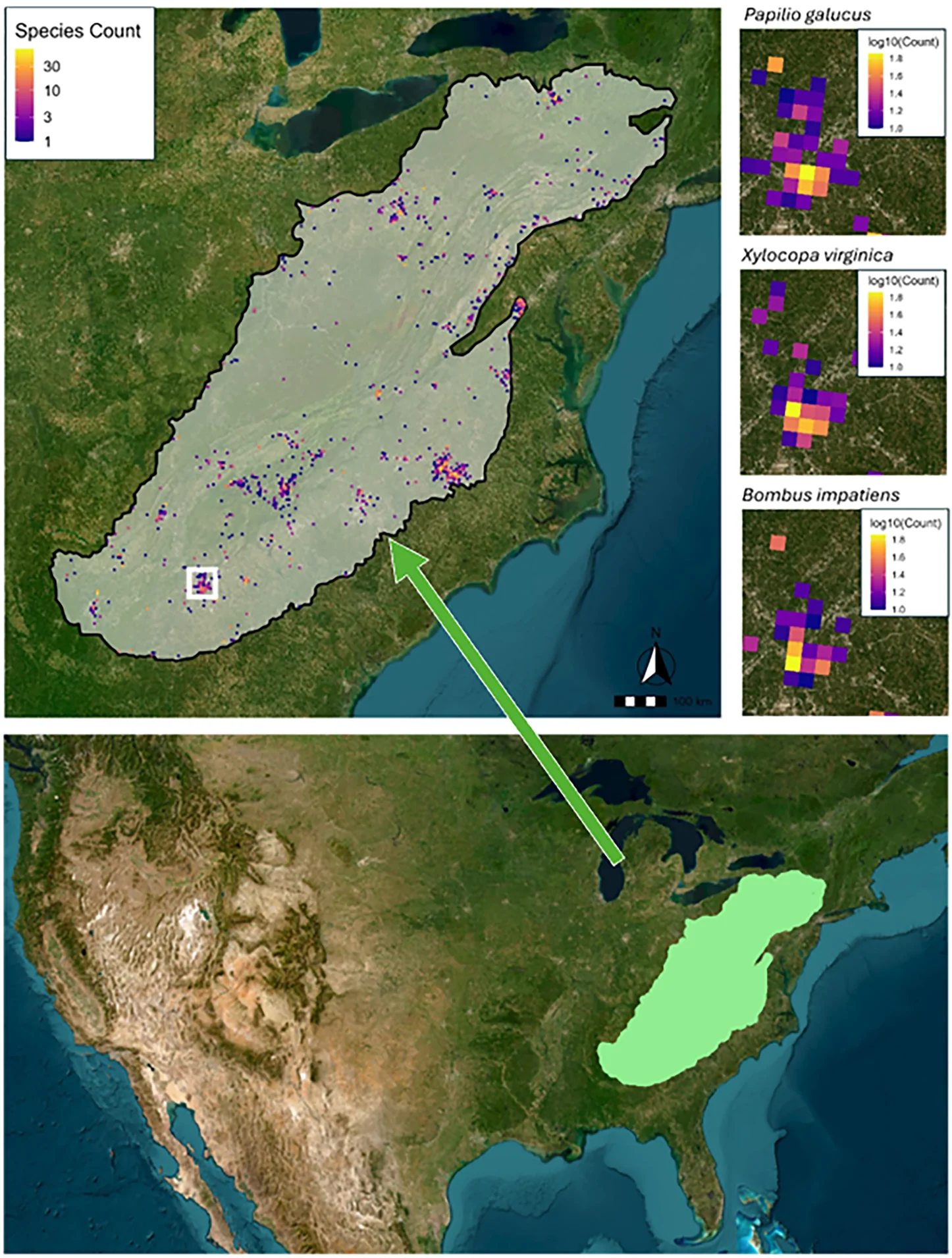
The number of species in each 5 × 5 km^2^ grid cell in Bioregion NA24 that met our filtering criteria. The panels on the right-hand side show variation in observation count by grid cell for three example species. These panels are a zoom into the city of Atlanta, Georgia, USA, denoted by the white square box, as an example of variation in observation counts.

Using the One Earth Bioregion Framework, we constrained data collection to the One Earth Appalachia & Allegheny Interior Forests bioregion, henceforth referred to as *Bioregion NA24*. A One Earth bioregion is defined by its biome(s), large-scale geographic features and climate zones [33]. We constrained our study to one bioregion so that we could limit the variation in climate zones and species composition. *Bioregion NA24* spans parts of the northeastern and southeastern USA (figure 1). Running along the Blue Ridge Mountains, this region spans from central Alabama to central New York. The dominant habitat type in *Bioregion NA24* is broadleaf forest, and it includes four ecoregions: Appalachian-Blue Ridge Forest, Appalachian Mixed Mesophytic Forest, Appalachian Piedmont Forest and Allegheny Highlands Forest. We chose this region because of its four distinct seasons and regular winter snowfall and freezes [34], and because it has variable levels of urbanization. This bioregion, which covers approximately 58.5 million hectares of land across 12 states, provides a large latitudinal spatial scale from which to examine phenology. It also encompasses major metropolitan areas such as Atlanta, Georgia (population 5.1 million) and Charlotte, North Carolina (population 1.38 million), highlighting the intersection of dense urban development with unique ecoregions [35].

### 2.2. Data collection

We obtained Research Grade iNaturalist data from the Global Biodiversity Information Facility (GBIF) for all species within the orders Coleoptera, Hymenoptera, Diptera and Lepidoptera and observations taken between 2008 and 2024 in a custom boundary around *Bioregion NA24* [36]. The Research Grade designation is given to observations that (a) meet iNaturalist data standards of a verifiable observation, (b) have acquired more than two-thirds agreement from the iNaturalist community on the taxonomic identification and (c) meet licensing requirements [37]. For this study, we defined pollinators as insects that engage in pollination activities (e.g. collect pollen/nectar and contact plants' reproductive parts). Using this definition, we created a manual literature search of adult diets to remove non-pollinator species observations from the GBIF dataset (see table 1 for a final list of species included in the study). For a species to be considered a pollinator, at least one credible source (peer-reviewed, published by a state or federal government or by a public university) had to state that the adults of the species consume flower nectar or pollen and have the body morphology to pollinate (e.g. body hair and functional mouth parts). Some ant species, for example, consume pollen but are not effective pollinators owing to their body size, which is not optimized to reach floral reproductive structures, their self-cleaning behaviour that removes pollen and their lack of flight dispersal [38]. Such species were removed from the study. Species were also removed from the study if no credible sources could be found about their adult feeding habits. We used this conservative definition of pollinators to ensure that the dataset would reflect pollinator phenology patterns and not the phenology of insects overall. We also used a manual literature search rather than an existing pollinator list so that the dataset would include species less widely known as pollinators. After filtering the pollinator observations to produce stable phenological estimates across an adequate range of Global Human Modification Index (GHMI) values (described below), we conducted this manual literature review. We removed 80 species from the study because they were not pollinators and 124 species because of a lack of information about their adult feeding habits (supplementary material, table S1). Many of the removed species were moths or beetles.

**Table 1.** Pollinator species included in the generalized additive model (GAM) analyses following literature-based verification of pollinator status and data-quality filtering (see Methods above), with summary statistics describing their coverage across the anthropogenic modification gradient (GHMI). Species are ordered by the number of grid cells with observations.

species	order	family	number of grid cells with observations	minimum GHMI	maximum GHMI	GHMI range
*Papilio glaucus*	Lepidoptera	Papilionidae	386	0.006	0.919	0.913
*Bombus impatiens*	Hymenoptera	Apidae	304	0.002	0.919	0.918
*Xylocopa virginica*	Hymenoptera	Apidae	222	0.006	0.919	0.913
*Danaus plexippus*	Lepidoptera	Nymphalidae	214	0.006	0.882	0.876
*Apis mellifera*	Hymenoptera	Apidae	182	0.006	0.919	0.913
*Epargyreus clarus*	Lepidoptera	Hesperiidae	137	0.006	0.919	0.913
*Atteva punctella*	Lepidoptera	Attevidae	100	0.162	0.919	0.757
*Dione vanillae*	Lepidoptera	Nymphalidae	100	0.002	0.919	0.918
*Battus philenor*	Lepidoptera	Papilionidae	98	0.000	0.88	0.88
*Phyciodes tharos*	Lepidoptera	Nymphalidae	94	0.006	0.846	0.84
*Junonia coenia*	Lepidoptera	Nymphalidae	85	0.006	0.919	0.913
*Elkalyce comyntas*	Lepidoptera	Lycaenidae	70	0.006	0.846	0.84
*Hylephila phyleus*	Lepidoptera	Hesperiidae	67	0.112	0.919	0.807
*Pieris rapae*	Lepidoptera	Pieridae	66	0.048	0.869	0.821
*Bombus griseocollis*	Hymenoptera	Apidae	64	0.006	0.869	0.863
*Pyrrharctia isabella*	Lepidoptera	Erebidae	60	0.048	0.831	0.782
*Lon zabulon*	Lepidoptera	Hesperiidae	59	0.336	0.846	0.51
*Papilio troilus*	Lepidoptera	Papilionidae	57	0.007	0.831	0.823
*Bombus bimaculatus*	Hymenoptera	Apidae	55	0.088	0.869	0.782
*Speyeria cybele*	Lepidoptera	Nymphalidae	54	0.006	0.831	0.824
*Papilio polyxenes*	Lepidoptera	Papilionidae	53	0.327	0.839	0.512
*Udea rubigalis*	Lepidoptera	Crambidae	44	0.156	0.831	0.675
*Bombus pensylvanicus*	Hymenoptera	Apidae	39	0.211	0.919	0.709
*Costaconvexa centrostrigaria*	Lepidoptera	Geometridae	39	0.156	0.808	0.653
*Scopula limboundata*	Lepidoptera	Geometridae	39	0.127	0.681	0.554
*Vespula maculifrons*	Hymenoptera	Vespidae	39	0.34	0.864	0.523
*Phoebis sennae*	Lepidoptera	Pieridae	36	0.008	0.919	0.911
*Coccinella septempunctata*	Coleoptera	Coccinellidae	35	0.007	0.864	0.857
*Euptoieta claudia*	Lepidoptera	Nymphalidae	34	0.006	0.839	0.833
*Toxomerus marginatus*	Diptera	Syrphidae	32	0.255	0.839	0.584
*Abaeis nicippe*	Lepidoptera	Pieridae	31	0.006	0.763	0.757
*Prochoerodes lineola*	Lepidoptera	Geometridae	31	0.064	0.792	0.728
*Toxomerus geminatus*	Diptera	Syrphidae	31	0.068	0.839	0.771
*Vanessa atalanta*	Lepidoptera	Nymphalidae	30	0.002	0.869	0.868
*Pleuroprucha insulsaria*	Lepidoptera	Geometridae	29	0.307	0.831	0.524
*Vanessa virginiensis*	Lepidoptera	Nymphalidae	29	0.112	0.763	0.651
*Calycopis cecrops*	Lepidoptera	Lycaenidae	27	0.241	0.808	0.567
*Alaus oculatus*	Elateridae	Coleoptera	26	0.068	0.808	0.74
*Lerema accius*	Lepidoptera	Hesperiidae	26	0.112	0.919	0.807
*Protographium marcellus*	Lepidoptera	Papilionidae	26	0.112	0.665	0.553
*Urbanus proteus*	Lepidoptera	Hesperiidae	26	0.112	0.919	0.807
*Tetraopes tetrophthalmus*	Coleoptera	Cerambycidae	25	0.048	0.839	0.79
*Chauliognathus pensylvanicus*	Coleoptera	Cantharidae	23	0.255	0.831	0.575
*Cisseps fulvicollis*	Lepidoptera	Erebidae	21	0.255	0.816	0.56
*Darapsa myron*	Lepidoptera	Sphingidae	21	0.13	0.67	0.54
*Polistes fuscatus*	Hymenoptera	Vespidae	21	0.308	0.869	0.561
*Scolia dubia*	Hymenoptera	Scoliidae	21	0.255	0.869	0.614
*Chauliognathus marginatus*	Coleoptera	Cantharidae	20	0.354	0.837	0.484
*Chlosyne nycteis*	Lepidoptera	Nymphalidae	20	0.006	0.808	0.802
*Hemaris diffinis*	Lepidoptera	Sphingidae	20	0.354	0.792	0.438
*Marimatha nigrofimbria*	Lepidoptera	Noctuidae	20	0.156	0.692	0.536
*Strymon melinus*	Lepidoptera	Lycaenidae	20	0.112	0.831	0.718

### 2.3. Phenological estimates

We used three different measures of pollinator activity: onset, offset and total duration. These are the beginning, end and duration of a species' season. We used iNaturalist observations of pollinators in *Bioregion NA24* to assess these phenological estimates within each grid cell. First, we used the R package *sf* to create a gridded map of *Bioregion NA24,* with 24 128 *5 × 5* km^2^ grid cells across *Bioregion NA24* [39]. The grid cell size that we used reflects the methodology used in [40]. We then collated the pollinator observations into their corresponding grid cells and filtered the dataset to include only grid cells where species had at least 10 observations. We did this to ensure that each species in each grid had enough observations to produce phenological estimates. After filtering the data, we used a Weibull probability distribution from the R package *phenesse* to produce phenological estimates for each species in each grid [41]. Using a method developed by [42], we measured phenology as: onset (10th earliest percentile of observations, i.e. beginning of season), offset (90th percentile of observations, i.e. end of season), and total duration (number of days in the season, quantified by subtracting onset from offset).

### 2.4. Organization of pollinator phenology across a human modification and climatic gradient

After estimating the phenology of each species in each grid cell, we assigned anthropogenic modification and climatic values to each grid cell. We used the GHMI dataset to quantify anthropogenic modification in each grid cell [43]. The GHMI dataset uses 13 individual datasets to estimate the proportion of a given area of land that is human-modified. It ranges from 0 to 1, with 1 being highly human-modified. Each GHMI pixel represents a 10 × 10 m^2^ area, and there are 250 000 pixels within a single 5 × 5 km^2^ grid cell. Using Google Earth Engine, we calculated the mean GHMI value of all pixels that fall within each 5 x 5 km^2^ grid cell in the dataset, creating a gridded measure of anthropogenic modification across *Bioregion NA24* that we combined with our pollinator observations and phenology estimates (figure 1). We also obtained daily temperature and precipitation data for each pixel in *Bioregion NA24* from Daymet V4 [44], accessed through Google Earth Engine, for the period 2008–2024. To estimate the average precipitation and temperature for each grid, we calculated the average daily minimum, average daily maximum and average daily precipitation per pixel, then calculated the average daily minimum, average daily maximum and average daily precipitation across all pixels in each 5 km grid cell.

**Figure 2.**
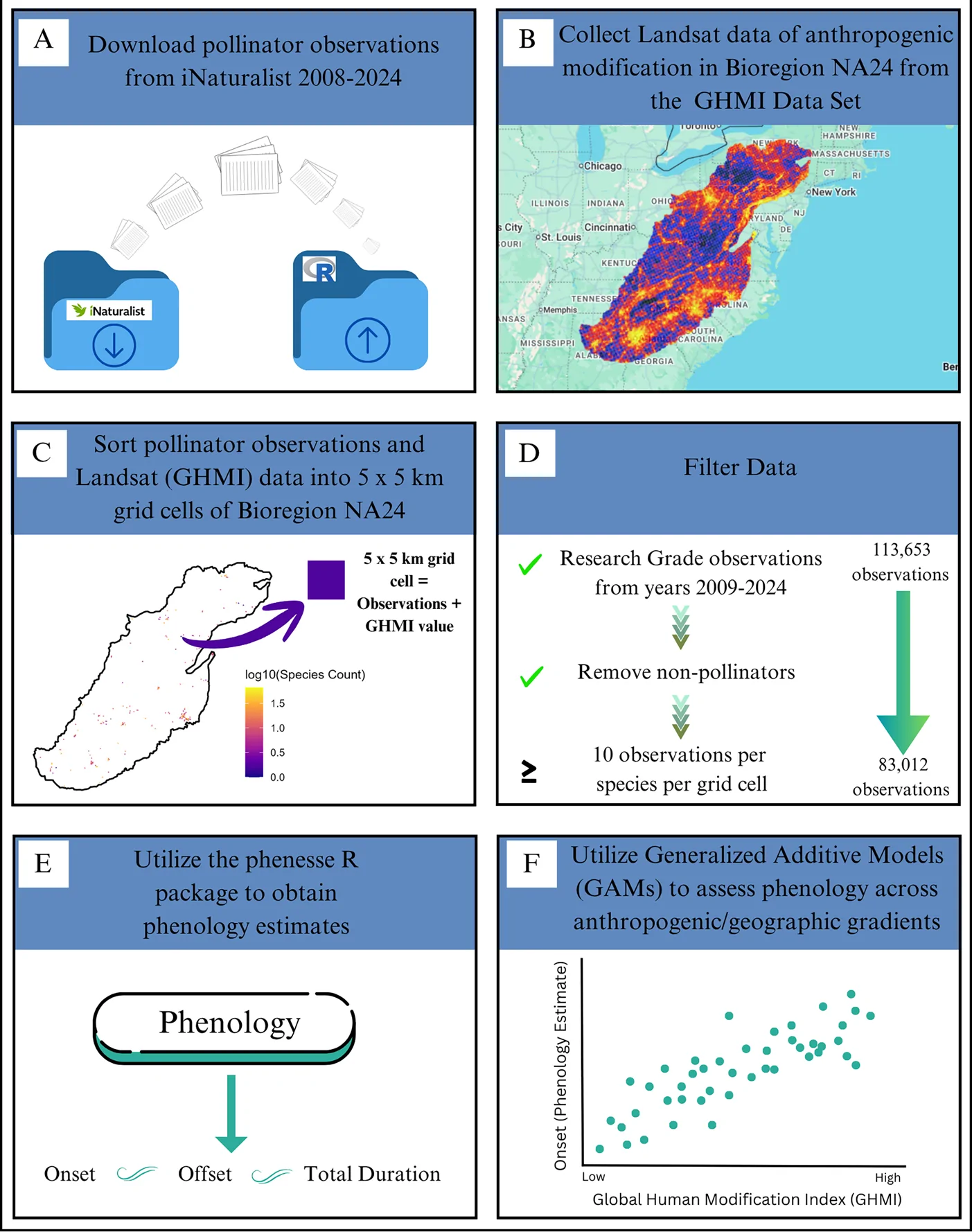
Step-by-step representation of the workflow used for data collection and analysis. We (A) downloaded photographic insect pollinator observations from Bioregion NA24 during the years of 2008 to 2024, (B) obtained the GHMI land cover data for Bioregion NA24, (C) created a gridded map of Bioregion NA24 and placed both observations and land cover data into the appropriate grids, (D) filtered the data for grid cells that had at least 10 Research Grade observations per species, (E) used the phenesse package in R ([41]; R version 2024.09.1) to calculate estimates of the beginning, end and total duration of activity periods for each species in each grid and (F) used GAM to test the significance of anthropogenic change as a predictor of the phenological estimates estimated in step E.

For modelling, the mean daily temperature was calculated as the average of the daily minimum and maximum temperatures for each grid cell. The final dataset for analysis included a range of grid cells across the eastern USA; each grid cell had a GHMI value, mean daily temperature value, mean daily precipitation value and phenological estimates (onset, offset and duration) for each species observed within that grid. To ensure that there were enough data and a wide enough range of GHMI values to model the relationship between phenology and GHMI, we only included species that have phenology estimates for at least 20 grids and that have a range and standard deviation of GHMI values of at least 0.30 and 0.10, respectively. See figure 2 for a full workflow of this methodology.

### 2.5. Data analysis

To answer our research objectives, we created a set of models to test whether anthropogenic modification significantly influences pollinator phenology. Specifically, we evaluated whether these relationships emerge at the regional level (i.e. across all species collectively) or at the species-specific level (i.e. for individual species). We organized our modelling framework into two categories: regional-level models and species-specific models (supplementary material, table S2). Within each category, we fitted three separate models corresponding to each phenological metric: (i) onset, (ii) offset and (iii) duration of pollinator activity. Regional-level models quantify the overall relationship between anthropogenic modification and phenology across all species, whereas species-specific models assess how these relationships vary among individual taxa.

### 2.6. Regional-level models

We fitted three separate regional-level models, where phenology responses were calculated across all species as a group. The three separate models were as follows: (i) regional-level onset, (ii) regional-level offset and (iii) regional-level duration. For each model, the predictor variables were GHMI, temperature, precipitation, species identity and geographic location (latitude and longitude). Because of the expected nonlinear relationship between phenology and the climatic and geographic variables, we used generalized additive models (GAMs) to model the data using the mgcv package in R [45]. We included GHMI as a linear predictor. Although we considered modelling GHMI as a smooth term, this resulted in worse model performance, as indicated by higher Akaike information criterion (AIC) values. Temperature and precipitation were modelled as smooth functions using thin-plate regression splines. We modelled geographic location (latitude and longitude) as a two-dimensional thin-plate spline of latitude and longitude [46]. In this way, we were able to assess the impact of human modification on phenology while accounting for spatial, taxonomic and climatic variation in phenology. To account for interspecific variation in phenology, we included species identity using a smooth with a random-effect basis. We compared these models to their associated null models (which did not have GHMI as a predictor) to assess whether GHMI improves model fit and increases the variation explained by the GAMs. We assessed multi-collinearity among predictors prior to model construction. While some moderate correlations were present (e.g. temperature and latitude), no pairwise correlations exceeded |*r*| = 0.9. In addition, the inclusion of spatial smooths reduces concerns about collinearity among spatially structured predictors. To determine the appropriate model family and smoothing parameter for temperature, precipitation and geographic location, we conducted model testing using gam.check() from the mgcv package in R [44] on several species with varying sample sizes, including one model that incorporated species as a random effect. Based on this, we fixed *k* at 20 for temperature, 20 for precipitation and 170 for geographic location to reduce the risk of overfitting for the species-specific models. For model diagnostics, we used the gam.check() function from the mgcv package [45], comparing response versus fitted values, histograms of residuals, residuals versus the linear predictor and deviance residuals against theoretical quantiles. Based on these diagnostics, as well as the distribution and type of response variable, we used a Gaussian distribution for all GAMs. All models were fitted using restricted maximum likelihood (REML) for more stable and conservative model estimates [47].

### 2.7. Species-specific models

Similar to above, we fitted three models for each species in the dataset, where each species was treated independently. The separate models were as follows: (i) species-specific onset, (ii) species-specific offset and (iii) species-specific duration. The model structure followed that of the regional-level models (i.e. the same predictor variables, treated the same way), with the only adjustment being that geographic location had a fixed *k* of 20, given the smaller sample size. All models were compared to a null model without GHMI as a predictor variable. Species sample sizes, measured by the number of occupied grid cells, ranged from 20 to 386. From these models, we examined the slope associated with GHMI and assessed whether the relationship was statistically significant.

### 2.8. Sensitivity and robustness analyses

We recognize that citizen science has biases that must be accounted for. In addition, the variables that we used for analysis and the structure of our data could produce misleading results. We, therefore, assessed these results and data structure in various ways. First, we assessed the extent to which GHMI (anthropogenic modification) mirrors urbanization in its relationship to pollinator phenology. To do this, we used population density, a commonly used proxy for human population density [48–50], from the gridded population of the world version 4.11 dataset (CIESIN 2018) in *Bioregion NA24* as an explicit measure of urbanization [51]. We used total population density for 2020 at 1 km resolution, averaged across 5 km^2^ grid cells. The resulting relationship between population density and phenology is very similar to the relationship between GHMI and phenology, suggesting that GHMI and urbanization similarly affect phenological estimates (see supplementary material, figs. S1 and S2).

Second, we recognize that there is a potential temporal mismatch between GHMI (a static variable produced in 2016) and the iNaturalist data, which are aggregated across a 16-year time span. However, the spatial grain that we used reduced the potential bias introduced by this temporal mismatch. The iNaturalist observations were aggregated into 5 km^2^ grid cells, and the measure of GHMI was at approximately 500 m resolution. Therefore, the potential of significantly different GHMI measures in a given grid cell over the course of the iNaturalist data aggregation is minimal. In addition, although the dataset spans a 16-year period, most observations are from more recent years, with the average observation year per grid concentrated between 2019 and 2024 with no strong relationship between average observation year and GHMI value (supplementary material, fig. S3). This was supported by a supplementary analysis, which showed that population density (which is produced every 5 years) shows very little change over the 16-year time span of the iNaturalist data (supplementary material, fig. S4). Further, we recalculated phenological estimates (onset, offset and duration) using a constrained dataset (only years 2012–2020; +/- 4 years of the 2016 measure) for the 10 most abundant species in the iNaturalist dataset. We found very similar grid-level phenological estimates, suggesting that these estimates are robustly measured in space (supplementary material, fig. S5).

Third, there may be temporal and spatial biases in sampling that could result in spurious correlations. With respect to temporal bias, we found that the distribution of sampled grid cells remained similar across years (supplementary material, fig. S6). However, the distribution of GHMI values across grid cells used in the analysis is uneven (supplementary material, fig. S7), raising the possibility that observed relationships may reflect an association between GHMI and sampling effort rather than a true phenological response to GHMI. To address this, we randomly resampled grid cells to ensure an equal number of observations within 0.2 GHMI bins, allowing us to assess the robustness of our findings. Although, this reduced the sample size from 758 to 433 grid cells, the relationships between mean GHMI and onset (estimate = 15.14, *p* = 0.681, adjusted *R*² = 0.50), offset (estimate = 17.046, *p* < 0.001, adjusted *R*² = 0.55) and duration (estimate = 2.879, *p* = 0.717, adjusted *R*² = 0.43) were similar to those from the full dataset (see regional-level results below).

## 3. Results

**Figure 3.**
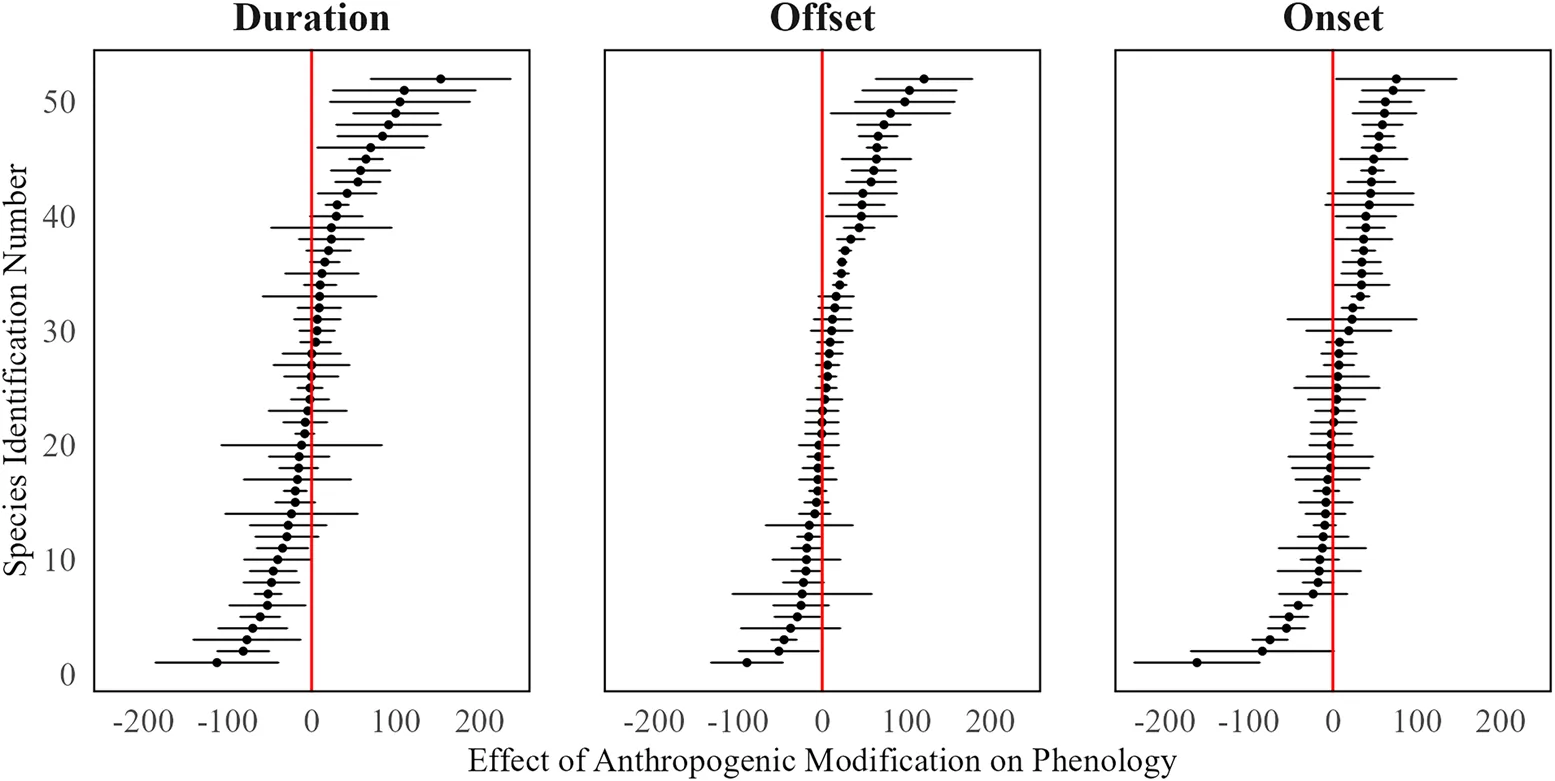
Slopes of species-level phenology estimates (total duration, offset and onset) across the gradient of anthropogenic change (GHMI). Each point represents the estimated slope from a species-specific GAM relating a phenological metric to GHMI, with species denoted by numerical IDs on the y-axis. Error bars show standard error (SE) of the slope estimates. Each species appears once in each panel: one slope for onset, one for offset and one for total duration. A positive slope indicates that species phenology is delayed or lengthened as the level of anthropogenic modification increases; a negative slope exhibits a phenological advancement or shortening as the level of anthropogenic modification increases.

There were 1032 pollinator species and 113 653 observations in the original dataset of all observations of Lepidoptera, Coleoptera, Hymenoptera and Diptera species in *Bioregion NA24* in the years 2008–2024. After filtering, the final dataset for analysis included 52 species and 83 012 observations (table 1 and figure 3). These species spanned 20 families and four orders: Hymenoptera, Lepidoptera, Diptera and Coleoptera (supplementary material, fig. S8). The greatest number of observations was made later in the year (July, August and September). There were 756 grids that met the filtering criteria for analysis (figure 1). The GHMI values in the dataset used for analysis ranged from 0.0002 to 0.9192 (mean = 0.4999, s.d. = 0.2025). Mean daily temperatures ranged from 6.98 to 18.21°C across all grids used for analysis (mean = 13.57°C, s.d. = 2.64°C) and mean daily precipitation ranged from 2.49 to 5.99 mm (mean = 3.76 mm, s.d. = 0.57 mm). Anthropogenic modification significantly shifted the activity periods of 19 species (table 2). Model results for all species in the dataset can be found in table S3 in the supplementary material. Pollinator onset, offset and total duration of activity exhibited strong spatial structure across *Bioregion NA24* (estimated degrees of freedom, henceforth referred to as edf = 42.86 to 94.67, *p*-value < 0.001), consistent with broad geographic gradients in phenology, and species identity explained substantial additional variation in onset, offset and total duration (edf = 49.33 to 50.11, *p*-value < 0.001).

**Table 2.** GAM outputs for species that have a significant relationship (*p* < 0.05) between GHMI and onset, offset or duration of activity period. For each species, we used phenology as a response variable and geographic location (latitude and longitude) as a two-dimensional thin plate spline. All models were run separately with onset, offset and duration as the response variables. Each model was compared to a null model, which did not include GHMI as a predictor variable. We present the species; number of grid cells with phenological estimates (sample size); model estimate (days per unit increase in GHMI), standard error (SE), *t* value and *p*-value of activity compared to anthropogenic change (GHMI); and adjusted *R^2^*, deviance explained and delta AIC.

onset									
			GHMI				model parameters		
species	order	sample size	estimate	SE	*t* value	*p*-value	adjusted *R^2^*	deviance explained	ΔAIC
*Papilio glaucus*	Lepidoptera	386	32.76	10.57	3.10	0.002	0.21	0.23	0.00
*Xylocopa virginica*	Hymenoptera	222	–74.76	21.01	–3.56	<0.001	0.11	0.15	0.03
*Danaus plexippus*	Lepidoptera	214	47.09	13.47	3.50	<0.001	0.16	0.22	0.00
*Apis mellifera*	Hymenoptera	182	–55.18	21.96	–2.51	0.013	0.39	0.44	0.00
*Battus philenor*	Lepidoptera	98	54.97	17.90	3.07	0.003	0.26	0.34	0.00
*Hylephila phyleus*	Lepidoptera	67	–52.01	22.73	–2.29	0.026	0.40	0.46	0.00
*Bombus griseocollis*	Hymenoptera	64	–41.15	16.42	–2.51	0.015	0.40	0.46	0.00
*Papilio troilus*	Lepidoptera	57	54.48	20.11	2.71	0.009	0.30	0.38	0.00
*Bombus pensylvanicus*	Hymenoptera	39	58.97	23.82	2.48	0.020	0.58	0.71	0.00
*Chauliognathus marginatus*	Coleoptera	20	36.60	13.81	2.65	0.028	0.88	0.95	19.67
Offset									
*Papilio glaucus*	Lepidoptera	386	23.49	5.16	4.55	<0.001	0.14	0.15	0.00
*Bombus impatiens*	Hymenoptera	304	20.87	8.05	2.59	0.010	0.13	0.14	0.00
*Danaus plexippus*	Lepidoptera	214	27.32	6.96	3.92	<0.001	0.46	0.48	0.00
*Epargyreus clarus*	Lepidoptera	137	22.80	8.83	2.58	0.011	0.17	0.22	0.00
*Battus philenor*	Lepidoptera	98	65.01	12.01	5.41	<0.001	0.32	0.35	0.00
*Phyciodes tharos*	Lepidoptera	94	34.03	16.45	2.07	0.041	0.08	0.14	0.00
*Hylephila phyleus*	Lepidoptera	67	–45.42	15.03	–3.02	0.004	0.12	0.19	0.00
*Pieris rapae*	Lepidoptera	66	43.96	18.23	2.41	0.019	0.05	0.13	0.00
*Pyrrharctia isabella*	Lepidoptera	60	73.45	31.82	2.31	0.025	0.06	0.14	0.00
*Scopula limboundata*	Lepidoptera	39	66.58	22.98	2.90	0.007	0.43	0.59	0.56
*Pleuroprucha insulsaria*	Lepidoptera	29	–89.52	42.69	–2.10	0.050	0.59	0.72	0.00
*Marimatha nigrofimbria*	Lepidoptera	20	61.33	26.23	2.34	0.035	0.11	0.34	0.00
Duration									
*Bombus impatiens*	Hymenoptera	304	30.43	13.60	2.24	0.026	0.10	0.13	0.00
*Xylocopa virginica*	Hymenoptera	222	64.66	20.14	3.21	0.002	0.29	0.34	0.00
*Apis mellifera*	Hymenoptera	182	55.11	26.84	2.05	0.042	0.30	0.35	0.00
*Papilio troilus*	Lepidoptera	57	–61.14	23.58	–2.59	0.012	0.20	0.28	0.00
*Abaeis nicippe*	Lepidoptera	31	–81.31	30.83	–2.64	0.016	0.59	0.73	0.00
*Chauliognathus marginatus*	Coleoptera	20	–51.79	16.06	–3.23	0.011	0.77	0.89	53.24

### 3.1. Onset

At the regional scale (regional-level models), GHMI showed strong evidence of a positive relationship with the onset of pollinator activity (*p*-value < 0.001, GHMI estimate = 22.12, adjusted *R^2^* = 0.53). Pollinator activity began approximately 22 days later in more anthropogenically modified areas than in fully natural areas, on average, after accounting for geographic and climatic variation and species-level differences. Temperature had a significant nonlinear effect on duration of pollinator activity (edf = 12.29, *p*-value < 0.001), but precipitation did not (edf = 1.01, *p*-value = 0.910). The full regional-level onset model explained 55.4% of the deviance in the onset and performed better than the null model without GHMI (ΔAIC = 0).

**Figure 4.**
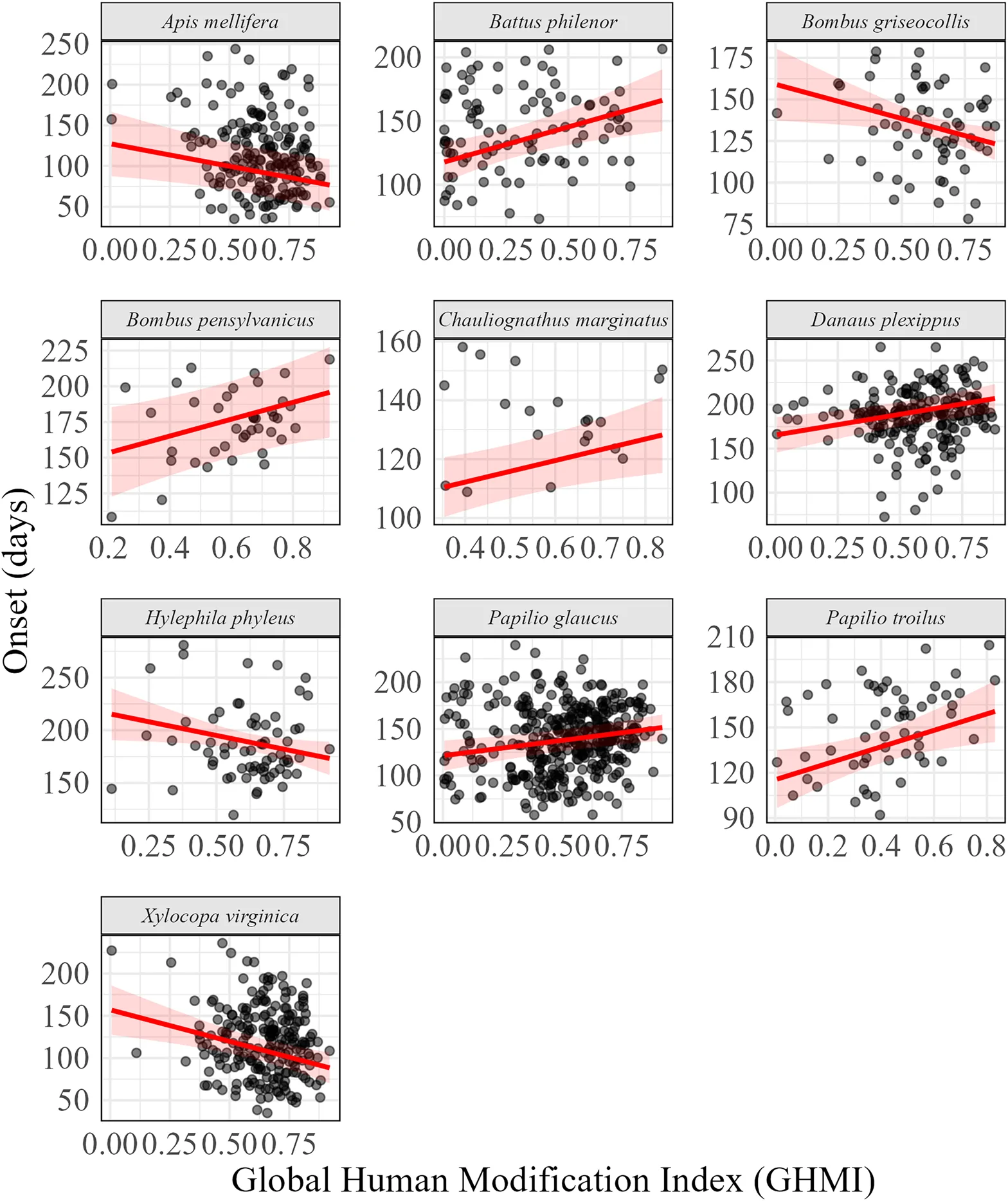
Observed and predicted onset values across the GHMI gradient for the 10 species whose species-specific GAMs indicated significant effects of anthropogenic change. Points indicate observed onset estimates, while red lines represent generalized additive model-predicted relationships. Onset estimates were computed with the phenesse package in R ([40]; R version 2024.09.1).

Of the 52 pollinator species used in the species-specific models, 10 species (19.23%) had a significant relationship (*p*-value < 0.05) between onset and GHMI (figure 4). Four Lepidoptera species, one Hymenoptera species and one Coleoptera species exhibited a delayed onset of activity in more anthropogenically modified areas (GHMI estimate = 32.76 to 58.97, adjusted *R^2^* = 0.16 to 0.88, deviance explained = 0.22 to 0.95; table 2). Alternatively, the onset of three Hymenoptera species and one Lepidoptera species was shown to begin earlier in more anthropogenically modified areas (GHMI estimate = –74.76 to –41.15, adjusted *R^2^* = 0.11 to 0.40, deviance explained = 0.15 to 0.46; table 2). When we compared the model with GHMI as a predictor with the null model for the significant species-specific models, GHMI models were the strongest supported models (ΔAIC = 0), except for one Hymenoptera species and one Coleoptera species (ΔAIC = 0.03 and 19.67, respectively). The average sample size (number of grid cells with phenology estimates) for the species-specific models that showed a significant relationship between GHMI and onset (mean = 135.0) was higher than the models without this significant relationship (mean = 49.0), indicating that some relationships may be obscured by small sample sizes.

### 3.2. Offset

The regional-level models showed strong evidence that GHMI has a positive relationship with the offset of pollinator activity at a regional scale (*p*-value < 0.001, GHMI estimate = 21.031, adjusted *R^2^* = 0.54). Offset of activity is approximately 21 days later in fully anthropogenically modified areas compared to fully natural areas after accounting for geographic, climatic and species-specific variation. The regional-level models showed no evidence that either temperature or precipitation had a significant nonlinear effect on offset of activity (edf = 2.015 and 1.00, *p*-value = 0.567 and 0.261, respectively). The full regional-level offset model explained 55.5% of the deviance in offset and performed better than the null model without GHMI (ΔAIC = 0).

Of the 52 species used in the species-specific models, 12 species (23.08%) had a significant relationship between offset and GHMI (supplementary material, fig. S9). Nine Lepidoptera species and one Hymenoptera species exhibited a delayed offset of activity in more anthropogenically modified areas (GHMI estimate = 20.87 to 73.45, adjusted *R^2^* = 0.05 to 0.46, deviance explained = 0.13 to 0.59; table 2). Two Lepidoptera species exhibited an earlier offset of activity in more anthropogenically modified areas (estimate = –45.42 and –89.52, adjusted *R^2^* = 0.12 and 0.59, deviance explained = 0.19 and 0.72, respectively; table 2). When we compared the significant species-specific models with the null models (which did not include GHMI as a predictor), most GHMI models were more strongly supported than the null models (ΔAIC = 0), with the exception of one Lepidoptera species (ΔAIC = 0.56). The average sample size for the species-specific models that had a significant relationship between GHMI and offset (mean = 126.0) was higher than the average sample size of the models that were not significant (mean = 47.4).

### 3.3. Total duration

Regional-level models exhibited no evidence that GHMI had a positive relationship with the total duration of pollinator activity (*p*-value = 0.863, GHMI estimate = 1.045, adjusted *R^2^* = 0.46). Eastern pollinator activity periods were less than one day longer on average in fully anthropogenically modified areas compared to fully natural areas, although this effect was not significant after accounting for geographic and climatic variation and species-level differences. Temperature had a significant nonlinear effect on duration of pollinator activity (edf = 8.759, *p*-value < 0.001), but precipitation did not (edf = 1.01, *p*-value = 0.771). The full regional-level duration model, which explained 47.7% of the deviance in total duration, did not perform substantially better than the null model without GHMI (ΔAIC = 0).

Six of the 52 species used in the species-specific models (11.54% of species in the dataset) showed a significant relationship between GHMI and the total duration of activity (supplementary material, fig. S10). Three Hymenoptera species showed a longer duration of activity in more anthropogenically modified areas (GHMI estimate = 30.43 to 64.66, adjusted *R^2^* = 0.10 to 0.30, deviance explained = 0.13 to 0.35; table 2). Two Lepidoptera species and one Coleoptera species had a shorter duration of the activity in more anthropogenically modified areas (GHMI estimate = –81.31 to –51.79, adjusted *R^2^* = 0.20 to 0.77, deviance explained = 0.28 to 0.89; table 2). Most of the significant species-specific models were more strongly supported than the null models with no GHMI (ΔAIC = 0), except for one Coleoptera species (ΔAIC = 53.24). The average sample size for the species-specific models that showed a significant relationship between GHMI and duration of activity was higher (mean = 136.0) than the average sample size of models that did not show significance (mean = 56.3).

Across all species-specific models, 10 species exhibited a significant change in more than one phenology estimate across a range of anthropogenic modifications. Four Lepidoptera species exhibited a shift in both the onset and offset of activity. Two Hymenoptera species, one Coleoptera species and one Lepidoptera species exhibited shifts in both the onset and duration of activity; one Hymenoptera species and one Lepidoptera species showed shifts in both offset and duration of activity (table 2).

## 4. Discussion

We found that pollinator activity periods in more anthropogenically modified areas start and end later in the year than those in less anthropogenically modified areas. This key finding suggests that pollinators as a group have shifted but not lengthened their window of activity to later in the year in areas of high human modification in the eastern USA. At a regional level, the onset of pollinator activity began approximately an average of 22 days later in the most human-modified environments, with activity seasons ending an average of 21 days later. By contrast, the total duration of pollinator activity showed weaker and more variable responses across taxa, indicating that pollinators do not uniformly shift the duration of activity in highly human-modified landscapes. Although this pattern was shown at a regional level for pollinators as a group, it was driven by Lepidopterans, which dominate the dataset (*N* = 36). Temperature also exhibited a strong nonlinear relationship with the duration and onset, but not the offset, of regional-level pollinator activity. This suggests that temperature change largely contributes to shifts in the onset of pollinator activity in highly human-modified areas, while other anthropogenic factors are driving shifts in the offset of activity. Together, these results are consistent with previous studies showing delayed pollinator seasons in urban environments with higher temperatures, highlighting the influence of anthropogenic modification on pollinator phenology [27,28,52].

This pattern contrasts with many studies of broad-scale climate warming, which often report advances in the onset of insect activity, particularly in spring-active taxa such as butterflies [53] and early-season bees [54]. These earlier onsets are typically attributed to accelerated degree-day accumulation, reduced winter severity and earlier attainment of thermal thresholds under regional or global climate change [55,56]. However, urban warming is distinct from broad-scale climate warming and interacts with other anthropogenic factors to compound impacts on phenology. For example, a study of Ohio butterflies showed that the onset of butterfly activity advanced under the pressure of warming and also advanced in urban areas, but was delayed in warm urban areas [52]. So, although the urban warming associated with anthropogenic modification may seem to mirror climate warming, it may shape pollinator phenology through distinct seasonal pathways.

The delayed offset and shifts in pollinator activity observed across an anthropogenic gradient probably reflect a combination of thermal and resource-mediated mechanisms associated with human-modified environments. We speculate that the urban heat island (UHI) could be a factor driving this pattern, as it elevates temperatures by 5–15°C relative to surrounding rural areas and prolongs conditions suitable for insect activity late into the year [57,58]. Urban warming also reshapes floral phenology, as some angiosperms exhibit extended or delayed blooming periods in cities, particularly late-season bloomers who experience extended flowering seasons [20,59,60]. In addition to thermal effects, human planting preferences further modify the seasonal availability of floral resources. Together, the UHI and human-driven vegetation changes could be creating altered resource windows that pollinators may be matching. This pattern could indicate a phenological mismatch of early-season pollinators in the eastern USA, which could alter the community structure and function of ecological communities in areas of high human modification [61–63]. While our supplementary analysis suggests that urbanization (as measured by population density) produces similar patterns to GHMI, we acknowledge that these mechanisms are speculative, as our study does not directly quantify UHI effects or plant phenology. Future work integrating climatic, vegetation phenological changes and pollinator data (e.g. [64–66]) will be critical for disentangling the relative contributions of these processes.

Although a delayed but not lengthened season was the dominant regional signal, our species-level analyses revealed differential responses in how pollinators respond to anthropogenic modification. While some pollinator species started and ended their seasons later in areas of high anthropogenic modification, others experienced an earlier start and end to their activity. Some species did change the length of their seasons. This variation was not consistent among orders. The dataset that we used was heavily dominated by Lepidopterans compared to Hymenoptera, Coleoptera and Diptera (*N* = 36, 9, 5 and 2 species, respectively; supplementary material, fig. S8). Of the models that showed phenology to be significantly predicted by anthropogenic modification, most Lepidopterans showed shorter and/or later seasons, most Hymenopterans showed delayed onsets and/or shorter seasons and the one Coleopteran species showed a shorter season with a delayed offset. Despite these general trends, there was species-specific variation in phenological responses within orders. These species-specific responses probably reflect differences in temperature-sensitive life-history traits that vary widely among pollinators, including overwintering stage (discussed in [67]), flexibility in diapause cues [68], voltinism [69] and thermal tolerance [70]. For example, multivoltine species may be better able to exploit extended warm seasons, whereas univoltine species with fixed diapause cues may be constrained in their seasonal timing [71]. Similarly, perhaps pollinators with higher thermal tolerances are more able to advance their seasonal onset in warm early-season UHIs, whereas species that require lower temperatures to initiate activity may delay the onset of activity. Urban warming, which increases soil temperature [72], may have a strong impact on pollinators that overwinter underground. Soil temperatures in high-altitude forests, for example, buffer the impact of warmer spring temperatures on the emergence of bumblebee queens [62,73]. The increased soil temperature in UHIs may remove this buffer and cause earlier spring-time emergence of bumblebee queens. Incorporating life-history traits into future models could help explain why some species lengthen their activity seasons at the early versus late ends, while others shorten or shift seasons.

The use of citizen science data allowed us to study the broad effect of anthropogenic modification on pollinators at large regional and taxonomic scales. However, this dataset is taxonomically, temporally and spatially biased. Lepidoptera were over-represented in our sample, and iNaturalist data have a strong May–September bias in the northern hemisphere [74]. In addition, iNaturalist data are more common in anthropogenically modified areas (supplementary material, fig. S8). The nature of the spatial arrangement of observations meant that we needed to take a coarse spatial grain for estimating human impact to have sufficient observations to measure phenological shift. These analyses may have missed finer spatial scale shifts in favour of uncovering broad patterns. In spatially heterogeneous cities, for example, areas of low human modification serve as refugia for pollinators [75,76], suggesting that combining our macroecological responses with fine-scale micro-scale analyses may help uncover a mechanistic understanding. In addition, a comparison of plant and pollinator phenology in this region is necessary to ascertain whether pollinator phenology shifts are driven by vegetation and to determine whether phenological mismatches are occurring.

Overall, we found that in the eastern USA, pollinators as a group in areas of high human modification have later but not extended seasons. Importantly, temperature has a stronger influence on the beginning than the end of pollinator seasons in areas of high human modification. Rather than a uniform shift across taxa, phenological responses varied among species, indicating that trait-mediated responses and plant-pollinator relationships probably shape how pollinators respond to human-modified landscapes. Accounting for this heterogeneity will be essential for predicting phenological mismatches and community reorganization as anthropogenic modification continues to expand.

## Supplementary Material



## Data Availability

All analyses were conducted in R version 4.4.2 [77]. Pollinator iNaturalist data were downloaded from GBIF [36]. The GHMI is available from Kennedy *et al.* [42] and was accessed via Google Earth Engine. All code and data to reproduce our analyses are available in an archived Zenodo repository [78]. Supplementary material is available online [79].
