# Supplementary material for: Anthropogenic modification shifts pollinator phenology in the eastern USA
Source: R Soc Open Sci. 2026 Sep 9;13(9):260267. doi: 10.1098/rsos.260267 (PMC13561042; doi:10.1098/rsos.260267)
Supplement: Supplementary file 1 [file rsos.260267.zip › rsos.260267/rsos260267_si_001.docx]

Title: Species Removed from the Study

Caption: **Table S1**. List of all insect species removed from the study because of a lack of credible information about adult feeding habits or because credible information about their adult feeding habits indicated that they are not pollinators.

Title: Models Overview

Caption: **Table S2**. Overview of generalized additive models (GAMs) used to assess relationships between anthropogenic modification and pollinator phenology.

Title: All Model Results

Caption: **Table S3.** Model results for all species in the study.

Title: Population Density versus GHMI

Caption: **Figure S1.** Relationship between species-specific slope estimates for (A) duration, (B) onset, and (C) offset in relation to anthropogenic modification (GHMI; x-axis) and population density (y-axis). Correlations between slope estimates from each predictor are shown within panels. Population density was derived from the Gridded Population of the World, Version 4.11 (GPWv4; CIESIN 2018), using total population density for 2020 (the closest year to the majority of observations), averaged across 5 km grid cells.

Title: Relationship between Phenology and GHMI versus Phenology and Population Density

Caption: **Figure S2.** The relationship between population density and phenology (A) versus the relationship between GHMI and phenology (B). Phenology is measured as the onset, offset, and duration of activity. The x-axis depicts the effect of population density (A) or GHMI (B) on phenology from species-level models. Each point is derived from the results of species-specific Generalized Additive Models, relating a phenological metric to GHMI or population density, with species denoted by numerical IDs on the y-axis. Error bars show standard errors of the slope estimates. Each species appears once in each panel: one slope for onset, one for offset, and one for total duration.

Title: Temporal Distribution of Grids, Observations, and GHMI

Caption: **Figure S3.** Average year that observations were taken per grid (A) and temporal distribution of observation data (B). Additionally, displayed is a comparison of mean GHMI value by mean year, which shows no strong relationship (Adj. R2 = 0.02).

Title: Temporal Distribution of Grid-Level Population Density

Caption: **Figure S4.** Change in population density from 2010 to 2020 for each grid cell used in the analysis, shown as individual blue lines. The black line represents the mean population density across all grid cells for each year. Population density was derived from the Gridded Population of the World, Version 4.11 (GPWv4; CIESIN 2018) and averaged across 5-km grid cells.

Title: Phenology versus GHMI for a Full and Subset of Data

Caption: **Figure S5.** Phenological estimates (A: duration, B: onset, and C: offset) across a range of anthropogenic modification (GHMI) for the top 10 most abundant species in the full years of iNaturalist data used in analysis (2008-2024) versus a subset of years (2012-2020). The phenological estimates produced from the subset of years are closer in time to 2016, which is the year from which GHMI estimates were produced. Phenological estimates were estimated at the grid-level. The distribution of grid-level estimates from 2008-2024 over a range of GHMI very closely resembles the grid-level estimates from 2012-2020 over this same GHMI range.

Title: Temporal Distribution of Observations over GHMI Range

Caption: **Figure S6.** Distribution of iNaturalist pollinator observations over a range of human modification (GHMI) from the years 2008-2024. Each plot shows the number of observations (y-axis) at each GHMI level (x-axis) for that particular year. The distribution of observations over a range of GHMI does not drastically change from year to year.

Title: GHMI Distribution Before versus After Filtering

Caption: **Figure S7.** Comparison of the distribution of GHMI values available in the study region (left) versus the distribution of GHMI values in that grid cells that were used in analysis (right). The iNaturalist pollinator observations used for analysis were biased towards areas of higher anthropogenic modification.

Title: Observations Across GHMI Range by Order

Caption: **Figure S8.** Observation frequency of pollinators, separated by order, in low versus high areas of human modification. Human modification was measured as the Global Human Modification Index (GHMI) value. Low human modification areas range from GHMI values of 0.0 - 0.3 and are shown in red. High human modification areas range from GHMI values of 0.7 to 1.0 and are shown in blue.

Title: Pollinator Offset Across a Gradient of Anthropogenic Modification

Caption: **Figure S9.** Observed and predicted offset values across the GHMI (anthropogenic modification) gradient for the species whose species-specific GAMs indicated significant effects of anthropogenic change. Points indicate observed offset estimates, while red lines represent GAM-predicted relationships. Offset estimates were computed with the phenesse package in R (Belitz et al., 2020a; R version 2024.09.1).

Title: Pollinator Duration Across a Gradient of Anthropogenic Modification

Caption: **Figure S10**. Observed and predicted total duration values across the GHMI (anthropogenic modification) gradient for the species whose species-specific GAMs indicated significant effects of anthropogenic change. Points indicate observed duration estimates, while red lines represent GAM-predicted relationships. Duration estimates were computed with the phenesse package in R (Belitz et al., 2020a; R version 2024.09.1).
